# Manipulation of photoluminescence of two-dimensional MoSe_2_ by gold nanoantennas

**DOI:** 10.1038/srep22296

**Published:** 2016-02-29

**Authors:** Haitao Chen, Jiong Yang, Evgenia Rusak, Jakob Straubel, Rui Guo, Ye Win Myint, Jiajie Pei, Manuel Decker, Isabelle Staude, Carsten Rockstuhl, Yuerui Lu, Yuri S. Kivshar, Dragomir Neshev

**Affiliations:** 1 Nonlinear Physics Centre, Research School of Physics and Engineering, Australian National University, Canberra, ACT 2601, Australia; 2 Research School of Engineering, College of Engineering and Computer Science, Australian National University, Canberra, ACT 0200, Australia; 3 Institute of Theoretical Solid State Physics, Karlsruhe Institute of Technology, 76131 Karlsruhe, Germany; 4 Institute of Nanotechnology, Karlsruhe Institute of Technology, 76021 Karlsruhe, Germany

## Abstract

Monolayer molybdenum diselenide (MoSe_2_), a member of the TMDCs family, is an appealing candidate for coupling to gold plasmonic nanostructures as it has smaller bandgap and higher electron mobility in comparison to frequently studied molybdenum disulfide (MoS_2_). The PL of MoSe_2_ occurs in the near-infrared spectral range where the emissive properties do not suffer from the enhanced dissipation in the gold due to inter-band transitions. Here, we study the interaction between monolayer MoSe_2_ and plasmonic dipolar antennas in resonance with the PL emission of MoSe_2_. By varying the thickness of the spacer between the MoSe_2_ layer and nanoantenna, we demonstrate manipulation of the PL intensity from nearly fourfold quenching to approximately threefold enhancement. Furthermore, we show that the coupled TMDC-nanoantenna system exhibits strong polarization-dependent PL, thus offering the possibility of polarization-based emission control. Our experimental results are supported by numerical simulations as well. To the best of our knowledge, this is the first study of Au-MoSe_2_ plasmonic hybrid structures realizing flexible PL manipulation.

Two-dimensional transition metal dichalcogenides (TMDCs) are likely to be important components for future optoelectronic applications due to their advanced properties^1^,^2^. A number of intriguing optical phenomena in TMDCs have been explored so far, including strong spin-valley coupling^3^,^4^, doping-dependent charged excitons^5^,^6^, piezoelectricity^7^, and valley coherence^8^. Additionally, novel integrated devices utilizing the properties of TMDCs have been recently demonstrated, including ultra-sensitive photodetectors^2^,^9^ and low threshold lasers^10^. In the latter, the interaction between TMDC and a photonic crystal cavity emphasizes the importance to control the emission from such 2D semiconductors through interactions with photonic nanostructures.

Localized plasmon resonances (LPR) sustained by metallic particles offer an important means to control the emission from quantum emitters due to high Purcell enhancement. As such, the hybrid systems of plasmonic nanoparticles with 2D TMDC materials have been a subject of intense interest. So far, research in hybrid systems composed of plasmonic nanostructures and TMDCs have mainly focused on MoS_2_. Effects such as photocurrent enhancement^11^, atomic-scale morphological detection^12^, and nanophotonic circuit composed of a single silver nanowire and MoS_2_ flake^13^ have been studied. The influence of plasmonic nanoantenna on the emission of MoS_2_ at about 680 nm have also been explored in several consecutive publications, including monolayer MoS_2_ PL enhancement by silica-gold nanoshells^14^, gold nanoantennas^15^, silver bowtie nanoantennas^16^, silver nanodisks^17^ or sandwiched between a nanopatch antenna and a gold film^18^. Enhancement in PL of up to 2000 times has been seen by combining plasmonic resonances at the pump and emission wavelengths^18^. Plasmonic enhancement of PL has also been explored in WS_2_ monolayers (PL at shorter wavelengths of ≈620 nm) by coupling to gold nanoparticles^19^. However, in a number of these experiments, the interband absorption peak of gold (500–600 nm) could likely have affected the enhancement effect. While there are other members in the TMDCs family with emission in the infrared range (away from gold enhancend interband absorption), they remained unexplored, such as MoSe_2_. Besides, most of these experiments demonstrated emission enhancement of TMDCs as a combination from excitation and emission processes. Compared with enhancement in the excitation process, enhancement in the emission is more robust as it does not depend on the pumping scheme, which is especially important for electronically pumped emitting devices.

Interestingly, Bhanu *et al*.^20^ reported on the inverse effect, namely PL quenching when investigating a Au-MoS_2_ hybrid system. They attributed the quenching to the charge transfer from the monolayer MoS_2_ to the gold nanoantennas through the resulting Schottky barrier of 0.4 eV. However, the quenching of PL emission from the monolayer should also happen at very small distances between the antennas and the TMDC due to the coupling of the emitted photons into non-radiative plasmonic resonances of the nanoantennas, *i.e.* dark plasmonic modes^21^. Nevertheless, the control of PL from 2D materials in the full range from PL quenching to enhancement by varying the coupling to the plasmonic antennas (through the variation of the spacing to the TMDC) remains so far not explored.

Here, we investigate the coupling between a MoSe_2_ monolayer and gold nanoantenna arrays mainly focusing on the emission process. PL manipulation from quenching to enhancement was realized by changing the thickness of a spacer used to spatially separate the 2D material and the antenna arrays. Numerical simulations support our observed phenomena and reveal the coupling mechanism in this hybrid system. MoSe_2_ is used here because it has some superior properties compared with well studied MoS_2_, though both are of similar structure. Firstly, MoSe_2_ exhibits a smaller bandgap, higher electron mobilities, higher internal quantum efficiency, and much narrower line width compared to the extensively studied MoS_2_ 22,23,24. These intrinsic characteristics imply different applications compared to MoS_2_. Secondly, few-layer MoSe_2_ possesses a nearly degenerate indirect and direct bandgap, which makes it more suitable for external modulation of bandgap and optical properties^22^. Thirdly, the direct bandgap of MoSe_2_ is close to the optimal bandgap value of single-junction solar cells and photoelechemical devices^24^. Importantly, MoSe_2_ has a lower Fermi energy level (4.4 eV)^25^, resulting in reduced charge transfer when in contact with gold. While MoSe_2_ is an appealing candidate for coupling to gold plasmonic nanostructures with PL in near-infrared range (away from gold’s enhanced dissipation due to interband transitions), this has remained unexplored to date.

## Results and Discussion

Metal particles, such as these used in our experiment, can generally alter the emission of TMDCs in two ways. Namely, enhancing the local field in the excitation process or the local density of states in the emission process, at the location of the nanoemitter. Whereas the first effect directly translates to an increase of the excitation rate, the latter modifies the spontaneous emission rate. Such modification of the spontaneous emission rate is commonly referred to as the Purcell effect^26^. Generally, modification of the spontaneous emission rate is a more robust way for controlling the emission of such TMDCs as it does not depend on the pumping scheme, which is especially important for emitting devices that are electronically pumped. However, placing a nanoemitter close to a metal particle also creates additional, non-radiative channels due to the dissipative nature of metals at optical frequencies. This basically happens when the emitter couples to the higher order plasmonic modes that are non-radiative in nature. When an emitter is located too close to the antenna, the non-radiative relaxation rates can dominate, spoiling the benefits of the radiative decay rate enhancement introduced by the plasmonic nanoantenna. This competing process in the weak pumping regime can be quantified by the quantum yield, defined as the ratio between the radiative and total decay rates. PL modification, the readily measurable quantity that is commonly used in experiments, thus relies on a combined consideration of the excitation rate enhancement and the quantum yield^21^,^27^,^28^,^29^,^30^,^31^,^32^,^33^. The goal of our experiments is to investigate these processes especially regarding the emission part in a system composed of plasmonic gold-bar nanoantenna, and a monolayer of MoSe_2_.

### Photoluminescence manipulation by gold antennas

**Figure 1:**
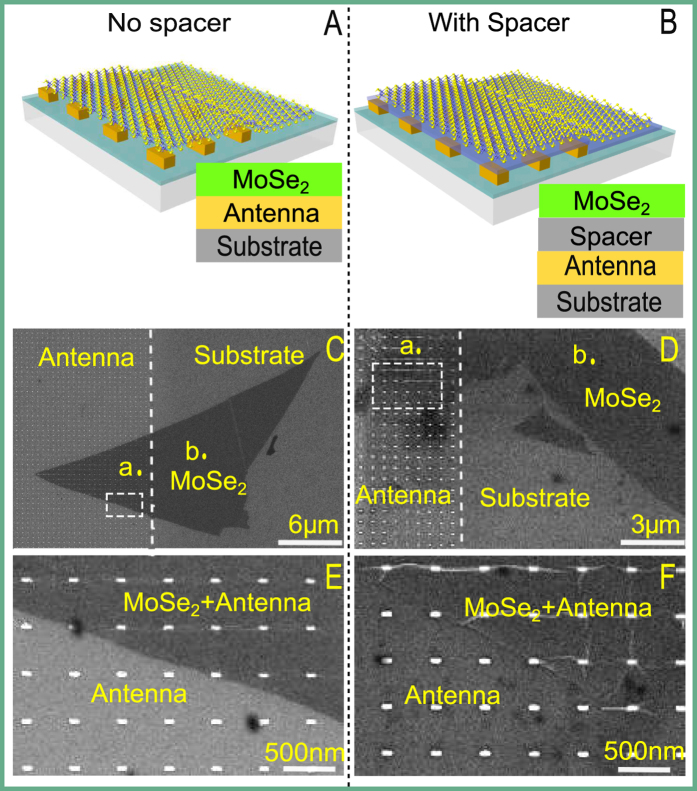
(**A**,**B**) Schematic side view of the two samples, without spacer and with spacer respectively. (**C**,**D**) The corresponding SEM images of the two samples used in experiments. PL spectra of spots (**a**) in the region of MoSe_2_-on-antenna and (**b**) in the region of MoSe_2_-on-substrate are shown and compared in Fig. 2C,D. (**E**,**F**) Magnified SEM images of the area bounded by dashed rectangles in (**C**,**D**) accordingly showing the shape of the antenna and how the monolayer flakes are positioned on antennas. The antenna length for the sample without a spacer is 127 nm and 100 nm for the sample with a spacer.

Rectangular gold nanoantennas with fixed width and height, both of 40 nm, and varying lengths in the range 70–130 nm were prepared (for details refer to the methods) and arranged in a square lattice with a center to center distance between adjacent elements of 505 nm. Such an antenna was chosen because their two different localized surface plasmon polariton can be selectively excited by using different optical polarizations. Next, single layer of MoSe_2_ samples were mechanically exfoliated from the bulk crystal and transferred onto the sample containing the plasmonic nanoantennas. The plasmon resonance of the antenna array is around the MoSe_2_ PL peak at ≈785 nm. At the same time, a second set of samples with slightly lower resonant wavelengths were coated with a thin layer silica spacer of 8.5 ± 1.5 nm through physical sputtering (the thickness is measured by an ellipsometer after the coating). The resonant wavelengths slightly red shifted due to the converage, because of the increase of the surrounding refractive index as experienced by the localized surface plasmon polaritons. This sample was designed such that the plasmon resonance spectrally coincides again with the the MoSe_2_ PL peak after application of the spacer layer. Then another piece of exfoliated monolyaer of MoSe_2_ was transfered onto the spacer-coated sample. For reference purposes, we let the monolayer flakes on both samples sit partly on the antenna array and partly on the silica substrate coated with a thin layer of indium tin oxide. The schematic pictures of our two samples are shown in Fig. 1A,B, respectively and the corresponding SEM images are given in Fig. 1C,D. Figure 1E,F show the gold nanoantennas and how the monolayer flake is positioned on the sample accordingly. The morphology of the TMDC on the nanoantennas was also tested by an AFM measurement in a non-contact mode, which reveals the conformal coating of the monolayer on top of the antennas.

**Figure 2:**
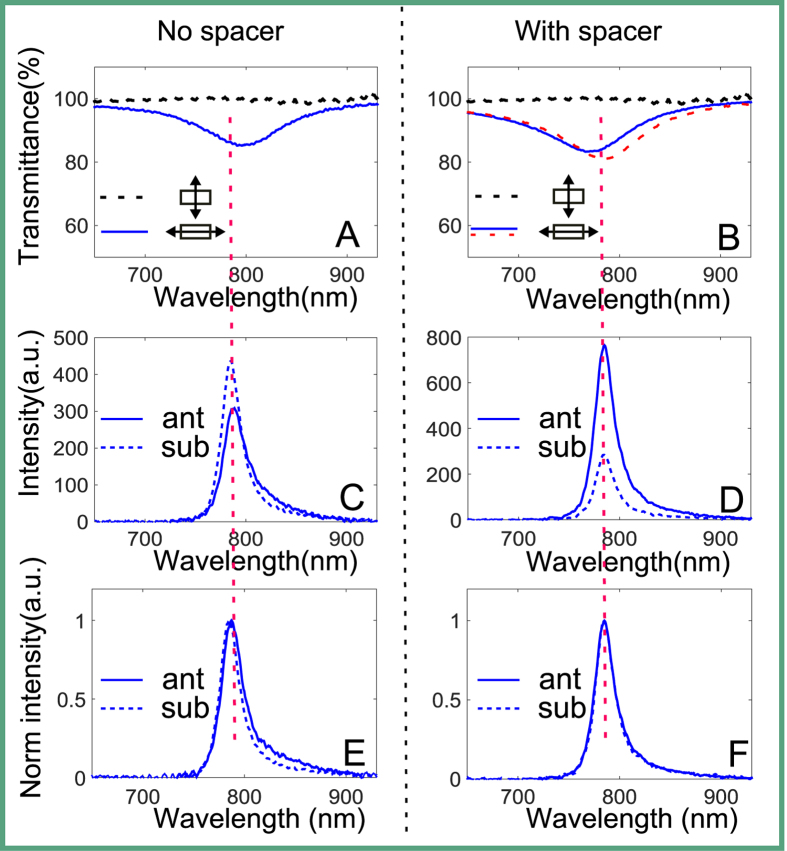
Spectral properties: (**A**) Transmittance profiles of the sample without spacer obtained by white light spectroscopy, the electric field of the illumination is polarized along the antenna long axis (solid blue line) and perpendicular to the long axis (dashed black line), respectively. (**B**) Transmittance profile of the sample with spacer before (blue line) and after coating the spacer layer (dashed red line), for illumination with its polarization along the antenna long axis. The transmittance for polarization perpendicular to the antenna’s long axis are identical in this spectral region before and after coating the spacer layer, as shown with dashed black line. (**C**) PL spectral profiles of the sample without spacer corresponding to points a (solid blue line) and **b** (dashed blue line) in Fig. 1C, the legend ‘ant’ here means on antenna and ‘sub’ means on substrate (same in the following). (**D**) PL spectral profiles of the sample with spacer corresponding to points a (solid blue) and b (dashed blue) in Fig. 1D. (**E**,**F**) Normalized spectra of (**C**,**D**) respectively.

The transmittance spectra of our antenna arrays are obtained by polarized white light spectroscopy and are shown in Fig. 2A,B. Here we distinguish the polarization of the incident illumination by its electric field that is either perpendicular or parallel to the long axis of the plasmonic rectangular nanoantenna. The spectra show pronounced transmittance dips near the central emission wavelength, marked with vertical dashed red line, when the polarization is set to be parallel to the long axis. Note that the contrast of the transmittance dips is not high due to the sparse arrangement of the antennas. Importantly, the antenna resonance disappears when changing the polarization of the incident illumination to be perpendicular to the antenna long axis (dashed black lines), confirming that the measured resonances are a consequence of the excitation of LPR in the nanoantennas. To make sure that the MoSe_2_ flakes used for our samples are monolayers, we measure their PL spectra excited by a 532 nm cw laser. The PL results are shown in Fig. 2C,D, which all show a good agreement with results for monolayer MoSe_2_ reported previously^22^,^34^, thus confirming that the flakes we used in experiments are monolayers. Furthermore, we have also conducted Raman measurements on the flakes excited by the same laser wavelenght. While less accurate than the PL identification, the measured first Raman peak of the flakes at 240.8 cm^−1^ (see Figure S3 of Supplementary Information) is consistent with the results for monolayer MoSe_2_ 22,34. Therefore, we could conclude that the MoSe_2_ flakes we used in experiment are monolayers.

Next we investigate the effects of the antenna on the spectral profiles of the monolayer MoSe_2_. The PL spectra of two typical regions, point a (in MoSe_2_-on-antenna region) and point b (in MoSe_2_-on-substrate region) (Fig. 1C,D), using excitation of 532 nm, are shown in Fig. 2C,D, respectively. The measured PL from the monolayer MoSe_2_ shows that the PL is quenched for the sample without a spacer, while it is enhanced for the sample with a spacer. Note that the absolute value of PL signal on MoSe_2_-on-substrate region in Fig. 2D (sample with spacer) is lower than its counterpart in Fig. 2C (sample with no spacer). This is likely due to the variation of the sample quality and not due to the spacer material. As alreday mentioned, our monolayer MoSe_2_ flakes were exfoliated from bulk crystal, hence the sample properties like size and formation may vary from one flake to another. As such, the PL signal could vary for different flakes. Besides, to obtain proper PL signal in experiments, we have further adjusted the excitation laser power accordingly for different samples. Thus, the comparison of the absolute PL values from different samples does not represent valuable information. Here we focus on the comparison of the PL on different parts of the same flake, which excludes variations of the PL signal from other factors like the sample quality. If the two samples we used in experiments were exactly the same, the PL value on MoSe_2_-on-substrate region shown in Fig. 2 would be the same. The normalized spectra are also shown in Fig. 2E,F accordingly. The flake on the sample without spacer shows slightly red-shifted and broadened spectrum on the antenna compared with the PL spectra on the substrate. The origin of the quenching effect cannot be unambiguously identified here, however it is likely due to the ohmic losses^21^ as well as possible additional charge transfer effect^20^, as the flake is in direct contact with the antenna. The changes of spectral position and intensity are relatively small compared to MoS_2_ 20, suggesting that the charge transfer is not as strong as in MoS_2_, which is supported by the larger Schottky barrier of 0.7 eV^20^,^25^. Therefore, we believe that the dominant reason for quenching is the increased non-radiative decay of the emission from the MoSe_2_ in close proximity to the gold antennas.

In contrast, the sample with a spacer shows almost the same spectral shape of emission for both MoSe_2_-on-antenna and MoSe_2_-on-substrate regions as shown in Fig. 2F, which is different from the results of broadened and red-shifted spectra reported in the literature^15^,^17^ about MoS_2_. This is a strong indication that the stronger PL on MoSe_2_-on-antenna region is mainly caused by emission enhancement and implies increased radiative decay due to plasmonic coupling in our system. Otherwise ohmic effects brought by the excitation enhancement will broaden and shift the spectral profiles^15^. Moreover, this unchanged spectra with enhanced intensity characteristic of this system is particularly beneficial for practical devices based on MoSe_2_ when considering its stability, as well as some optical applications that require stationary spectrum such as interferometry.

**Figure 3:**
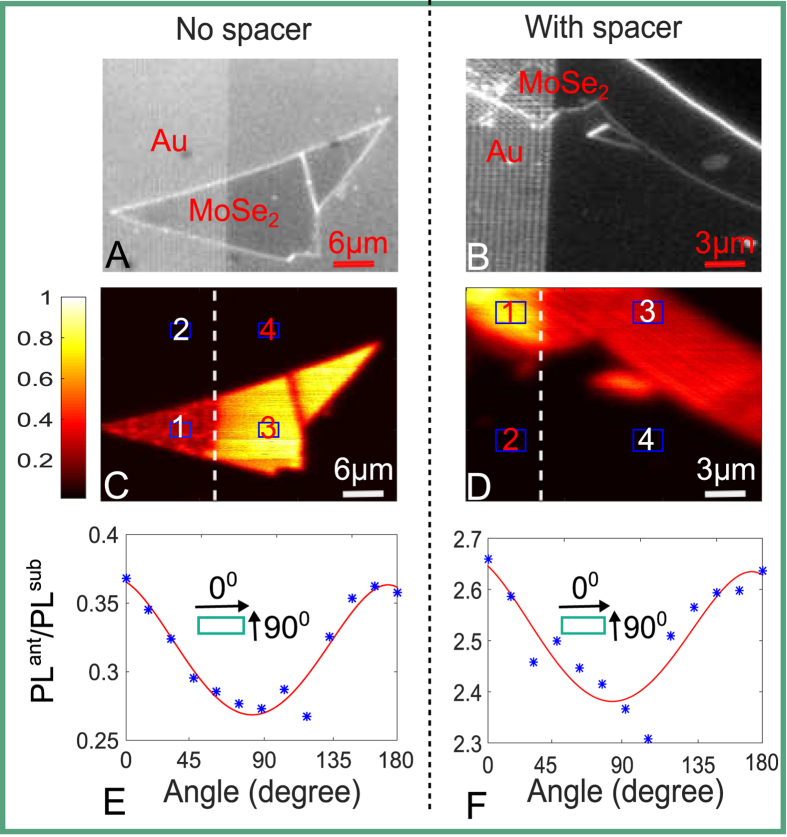
(**A**,**B**) Optical images of two samples, without a spacer and with a spacer, respectively. (**C**,**D**) Corresponding typical PL mapping images of the samples integrated over the range 715–1095 nm, both images are normalized to their respective maximum value. (**E**,**F**) Corresponding antenna effects varying with collection polarization angles of the two samples, measured data (asterisks) and fitting curves (red solid lines). The insets in (**E**,**F**) indicate the direction of polarization with respect to the gold rectangular antenna.

To characterize the influence of the antenna on the PL in more detail, we map the 2D PL image integrated over the spectral region 715–1095 nm. The optical images of the two flakes are shown in Fig. 3A,B, accordingly and the results of PL mapping are shown in Fig. 3C,D (both images are normalized to the corresponding intensity maximum), respectively. Both, the quenching effect for the sample without spacer and the enhancement for sample with spacer can be clearly seen there. We can also see that the emission from the gold antenna or the substrate regions is negligible as compared to the region covered by the monolayer MoSe_2_ flakes, which means the background is negligible.

In the following, we define the quantitative PL change due to coupling with antenna, called here antenna effect, as PL^ant^/PL^sub^. This quantity is calculated by taking the average PL value of a small area from the MoSe_2_-on-antenna region then subtracting the corresponding background and normalizing to the PL from the same area at MoSe_2_-on-substrate region, namely (①-②/③-④), as shown in Fig. 3C,D. It can be seen from the definition that the antenna effect would be greater than 1 for PL enhancement while less than 1 for quenching. We also collect the PL at different polarizations by adding a polarizer in the collection pathway and obtain the antenna effect varying with polarization angles, as shown in Fig. 3E,F for both samples, respectively. We observe approximately threefold maximal enhancement of the PL from the MoSe_2_ monolayer for the sample with spacer, while nearly up to fourfold quenching for the sample with no spacer. The antenna effects are obviously polarization dependent for both samples due to the excitation of the dominant LPR along the long antenna axis. We note that in our measurements we cannot clearly distinguish the individual antenna spatially, hence our measurements represent the average quantity of enhancement across the entire unit cell of the antenna array. This averaging reduces the effective enhancement/quenching effect, which can be much greater for some positions of the emitters (see the Numerical Modeling section below). Besides, the strongest enhancement effect happens when the polarization is along the antenna’s long axis, which reflects the intrinsic property of LPR resonance along the long axis.

To further investigate the interaction mechanisms of these hybrid systems, we measured the antenna effect variations with respect to different pumping wavelengths (see Supporting Information, Figure S2) using a supercontinum laser tunable in the range of 530–640 nm. No significant dependence was found in the PL emission by varying the excitation wavelength by more than 100 nm. This excitation wavelength insensitivity implies further that the observed phenomena are mainly induced by interaction between the antenna and MoSe_2_ during emission process, and the excitation does affect our measurements much. This behavior is confirmed in our numerical modeling part below.

### Numerical modeling

To support our experimental results and further understand the nature of the involved processes, we perform numerical calculations. The numerical calculations also allow us to see the effects of fine variation of the spacer thickness, which is not possible in our experiments due to the percolation of the dielectric spacer on top of the antenna for small thicknesses (<6 nm). To analyze the emission process, the interaction of an electric dipole emitter and the antenna is considered in the weak coupling regime. We perform calculations for both cases: once for an emitter coupled to the gold antenna and once for an emitter on a glass substrate for reference. To study the excitation process, the setup with and without the antenna is irradiated with a plane wave at a wavelength of *λ* = 532 nm. The polarization of the electric field is set to be parallel to the short axis of the antenna, corresponding to our experimental arrangement. We solve numerically Maxwell’s equations and obtain spatially resolved electro-magnetic field at the excitation wavelength (see the Methods section for more details). Equating the results for the excitation and the emission processes once in the presence of the nanoantennas and once in the referential situation allows for direct comparison with our experimental measurements as the method used in Ref. 21. All geometrical details considered here are consistent with the experiments.

In slightly more detail, the normalized excitation rate can be calculated as


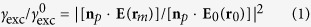


where **n**_*p*_ is the unit vector pointing in the direction of the dipole moment and **r**_*m*_ is the location of the dipole emitter. The fields **E**(**r**_*m*_) represent the induced electric fields at the location of the dipole emitter in a setup with antenna under plane wave illumination, and **E**_0_(**r**_0_) is the referential induced electric field on the surface of the substrate in a setup without antenna under same illumination. The emission is a result of the exciton recombination, which restricts the electric dipole moment of the emitter to be in the 2D plane of the MoSe_2_ flake. However the orientation of the dipole moment in this plane is uncertain^35^. We therefore consider an average ‘in-plane’ amplitude of the excitation field at *λ* = 532 nm and use


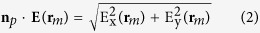


in the calculation of the field enhancement.

To characterize the emission process, we then calculate the quantum yield. This quantity is a measure for the quality of the antenna, since it accounts for the non-radiative and internal losses of the hybrid antenna - dipole emitter system. The normalized quantum yield is given by^36^


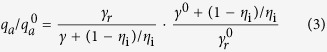


where *γ*_*r*_ and *γ* are the radiative and the total decay rate of the emitter, respectively, evaluated at the emission wavelength of *λ* = 785 nm. The superscript ‘0’ indicates the quantities calculated without the antenna. The quantity *η*_*i*_ is the intrinsic quantum yield of the dipole emitter^21^,^36^. For a perfect emitter, it holds *η*_*i*_ = 1 and the antenna can only reduce the overall system efficiency. For emitters with low *η*_*i*_, the overall radiative efficiency can however be effectively increased. For our simulations, we considered *η*_*i*_ = 0.05 to reflect the fact that MoSe_2_ is a rather poor emitter. This quantity is estimated by comparison of the experimental emission from flakes of MoSe_2_ and MoS_2_ 1 on a glass substrate at the same excitation power. The MoSe_2_ flake shows approximately an order of magnitude higher PL intensity, therefore we estimate its internal quantum efficiency to be a factor of 10 higher than MoS_2_ 1. Note that the quantum yield we used here is just a rough estimation and the actual value is unknown so far, eventually we wish to emphasize that a monolayer of MoSe_2_ is a rather poor emitter. However, the actual value we assume for the intrinsic quantum yield is of secondary importance considering the fact that a quantitative comparison to the experimental results is not our purpose. This would require spatial averaging of the emission process to reflect the fact that a monolayer MoSe_2_ covers the entire sparse antenna. And the detailed discussion of quantum yield of monolayer MoSe_2_ is beyond the scope of this work. In contrast, here we just want to reveal the physical mechanism behind our observed phenomena. Further details on the calculation of the remaining quantities can be found in the Methods section.

Finally, the fluorescence rate is a product of the normalized excitation rate 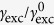 and the normalized quantum yield 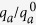 and is the quantity measured in the experiment:


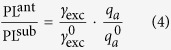


**Figure 4:**
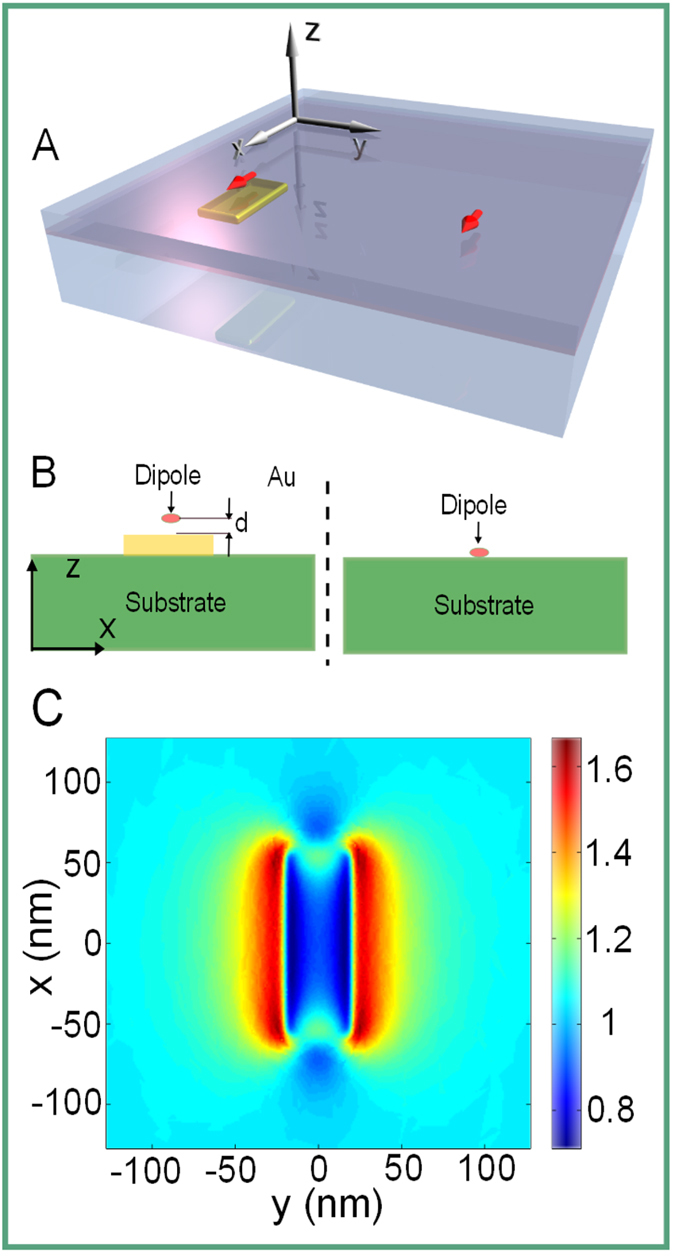
(**A**) Schematic of the model structure. On the left we show a dipole emitter (red arrow) above a metallic nanoantenna where the distance has been controlled with a spacer. On the right we show the reference situation that is geometrically the same except the antenna is missing. (**B**) XZ cross section of the simulation setup, *d* indicates the spacer thickness, the coordinates are consistent with the one shown in (**A**). (**C**) The enhancement of the electric field by the antenna, normalized to the reference case, calculated in a XY plane 7 nm above the antenna. The antenna (top view) is excited by a plane wave of the wavelength *λ* = 532 nm, polarized parallel to its short axis. The strongest field enhancement appears at the edges of the antenna.

A sketch of the considered geometry is shown in Fig. 4A. Figure 4B shows the cross section of the simulation setup as well. The enhancement of the electric field at the excitation wavelength of *λ* = 532 nm by the antenna in the plane where the MoSe_2_ flake locates in experiemnts is displayed in Fig. 4C. Here, the antenna (top view) is excited by a plane wave. Note that the strongest field enhancement appears at the edges of the antenna.

**Figure 5:**
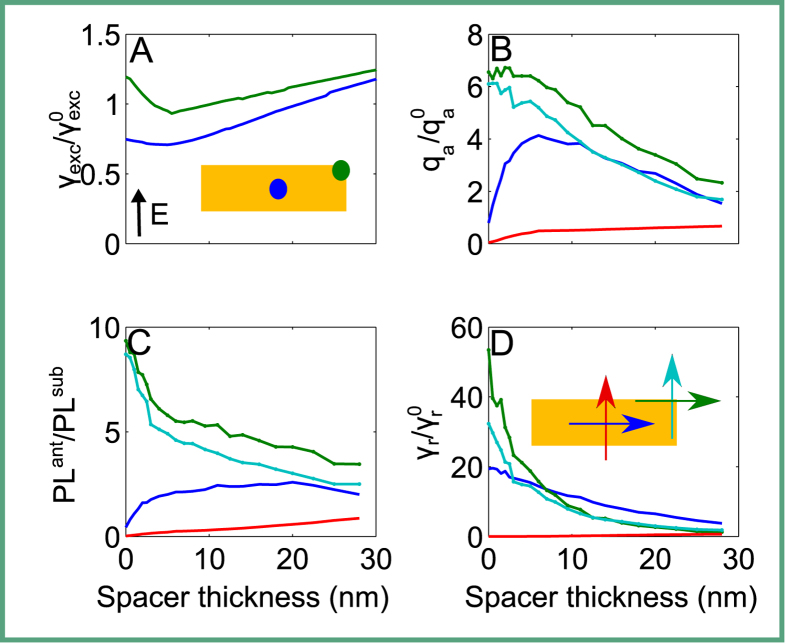
(**A**) The excitation rate of the electric field of a plane wave in the characteristic points of the antenna (center [dark blue] and a top-end corner [green]), normalized to the intensity of a plane wave in a setup without the antenna. The illumination plane wave is polarized along the short axis of the antenna as indicated by the inset arrow. (**B**) quantum yield, (**C**) fluorescence enhancement, and (**D**) radiative decay rate enhancement for a dipole emitter placed above the center of the antenna and polarized parallel to its long (dark blue) or short (red) axis; the quantities were calculated also for the case where the dipole emitter was placed above the top-end corner of the antenna and parallel to its long (green) or short (light blue) axis.

In the experiment, the transition dipole moments of MoSe_2_ are spatially distributed in the plane and the eventual measurement signal is the result of an ensemble averaging from all the individual positions and orientations as mentioned previously. Full numerical consideration of such averaging is resource consuming. Furthermore, the averaging will not provide a physical insight on the different mechanisms of PL modifications. Therefore, we chose to provide a qualitative understanding of the experimental results by studying the interaction of emitters placed at a few individual positions, as shown below. For this purpose, we consider two emitter positions relative to the antenna: one at a central position and one at a corner. In the following, we study the dependence of different physical quantities on the spacer thickness, *i.e*. the distance between the dipole emitter and the antenna. For simplicity and without loss of generality, we use vacuum as spacer material. The electric field intensity of a plane wave for the case with and without the antenna for both locations is shown in Fig. 5A. Please note that the excitation enhancement rate shows weak trend that it increases with increasing spacer thickness in the figure, this is caused by interference between incident field and the back reflection from the substrate, actually this quantity is always around unity for larger spacer thickness beyond the range shown in the figures. In full agreement with our experiments, the influence of the antenna on the excitation rate is weak. A minor dependency remains and is taken into account, but eventually the excitation field is not significantly modified when compared to the reference case. This is because the excitation wavelength is below the plasmonic resonance sustained by the antenna and no notable interaction is expected nor encountered.

The quantum yield, defined via Equation (3) is displayed in Fig. 5B and the fluorescence enhancement PL^ant^/PL^sub^ is shown in Fig. 5C. We could see from Fig. 5B that the quantum yield is enhanced and decreases with increasing spacer thickness when dipoles are at the corner of the antenna for both orientations. When dipole is positioned at the center of the antenna, the behaviors of the dipoles with different orientations are different. For the dipole oriented along the short axis of the antenna, the quantum yield is quenched and changes slightly with increasing spacer thickness. For the dipole orientated along the long axis of the antenna, the quantum yield is quenched when the dipole is very close to the antenna. Then the quantum yield increases with increasing spacer thickness until reaching its peak value at a spacer thickness around 7 nm. Afterward, the quantum yield starts dropping and asymptotically reaches unity. As discussed above, the quantum yield takes the leading role that affects the fluorescence rate which corresponds to the quantity we observed in experiments. So the fluorescence rate shown in Fig. 5C preserves the trends of the quantum yield with increasing spacer thickness except that the curves are flattened a little bit due to the multiplication with the excitation rate. Since the experimental results are a consequence of ensemble measurements, we could conclude that the quenching effects dominate for the sample in the absence of a spacer. In contrast, for a spacer with a finite thickness the enhancement of the fluorescence can be harvested. We could also infer from the simulation results that the optimum spacer thickness for enhancing the PL is around 7 nm (close to the value we used in experiments). Increasing further the space thickness will not result to more PL enhancement as the emitters interacts weakly with the antenna. Considering the fact that actually a large share of emitters will not be exposed to a spatial region in our experiments where the quantum yield is enhanced, the increase in the fluorescence signal by a factor of three (observed in our experiments) is quite remarkable.

The radiative decay rate enhancement 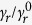 is often discussed as the measure for the gain of light that the dipole emitter will radiate into the far-field when coupled to the antenna^37^. The radiative decay rate enhancement for our antenna is shown in Fig. 5D. Enhancements by approximately up to two orders of magnitude can be seen. This enhancement in the radiative rate is eventually the reason for the observed increase in the fluorescence rate. Actually, the excited emitter has multiple decay channels. First of all, a very likely path for its de-excitation is the internal non-radiative recombination of the excitons and therefore non-radiative relaxation due to the low internal quantum yield. This quantity cannot be affected by the modified optical environment. Additionally, the dipole emitter can decay through radiative or non-radiative processes via the antenna. Crucially, these are the transition rates that are improved by the plasmonic antenna. The non-radiative decay due to the antenna is certainly undesirable, but it is a price that must be accepted to improve the radiative decay rate.

## Conclusion

We have studied the coupling of monolayer MoSe_2_ with plasmonic nanoantennas and have demonstrated emission manipulation in such materials from quenching to enhancement mainly through affecting the emission process. This manipulation is achieved by adding a dielectric spacer between the antenna and MoSe_2_ monolayer. Our experimental results are supported by numerical calculations, which further reveal the coupling mechanisms between the plasmonic antenna and MoSe_2_ monolayer. In particularl, we have observed that the nanoantenna enhances the radiation rate when compared to other non-radiative decay processes, *i.e.* especially the internal non-radiative decay. To harvest this positive aspect of the nanoantenna requires however to enforce the distance between the MoSe_2_ and the nanoantenna since otherwise quenching would dominate the processes. This has been clearly seen in our experiments and the observation is fully supported by the numerical simulation. To the best of our knowledge, the present work provides the first study of Au-MoSe_2_ system, offering more insights into the interaction between the nanoantenna and monolayer MoSe_2_. Importantly, MoSe_2_ is a largely unexplored member of the TMDC family, offering several advantages in comparison to its widely studied MoS_2_ and WS_2_ counterparts. Moreover, the enhanced PL with unchanged spectrum shape is meaningful for practical MoSe_2_ applications when considering its spectral stability. Furthermore, PL manipulation in our experiments is realized by affecting the emission process of MoSe_2_, this method is more robust as it is independent from the excitation scheme, which is especially important for devices with electrical pumping. Besides, enhancement (quenching) effect varying with excitation wavelength is also studied. The method presented here in general offers an important way for PL manipulation in large dynamic range from quenching to enhancement for these advanced materials, as well as the opportunity of polarization-based PL control, both of which are promising for future optoelectronic applications and developments.

## Methods

### Antennas fabrication and MoSe_2_ transfer

The nanoantennas were prepared by standard electron beam lithography (EBL). A 10 nm indium tin oxide (ITO) was coated by physical sputtering on the glass for conducting and adhesion purposes before we start our lithography process. PMMA950 photo resist has been used in the EBL process. Rectangular antennas, with fixed width and height both of 40 nm, and varying length from 70 nm to 130 nm were fabricated. After characterizing the transmittance spectra of these samples, some samples are coated with a thin layer silica by physical sputtering. The layer thickness was measured by ellipsometery.

Single layer of MoSe_2_ samples were mechanically exfoliated from the bulk crystal and drily transferred onto the substrate covering both the Au antenna arrays and adjacent substrate.

### PL-mapping

Micro-PL spectroscopy and micro-PL spatial mapping have been performed using a commercial WiTec alpha300S system in scanning confocal microscope configuration (for further details see Supplementary Information, Figure S1). For excitation, light from a supercontinuum laser with 5 nm spectral bandwidth tunable in the range 530–640 nm is focused on the sample with a 100× objective (NA = 0.9) from the MoSe_2_ side. The measured spot size of the excitation beam is ~1 *μ*m at 532 nm wavelength. The MoSe_2_ PL is then collected from the substrate side of the sample using a 50× (NA = 0.65) objective (transmission mode). A linear polarizer inserted into the detection path allows for selectively collecting the PL for different polarizations. In order to remove the light of the exciting laser source from the signal, a 715–1095 nm bandpass filter has been introduced. The spectrometer is fibre-coupled to an Ocean Optics spectrometer using a multimode (non-polarization maintaining) fibre to neutralize any possible polarization sensitivity of the gratings. To further rule out any unwanted effects from possible gold PL, we have tested the PL from base gold antennas, which was found to be below the noise level of our detection. For spatial mapping, the MoSe_2_ has been excited with an average power of 0.5 *μ*W, leading to an excitation power density of 637 W/cm^2^ on the sample. The PL has been collected for different polarizations using an avalanche photodiode in combination with a longpass filter.

### Numerical calculations

The numerical calculations were performed using a Finite Element Method (FEM) solver, as implemented in the commercially available software package COMSOL. The simulations were performed with open boundary conditions. To analyze the emission properties, an electric dipole emitter is placed in the computational domain and its emitted field has been calculated everywhere in space. The radiative decay rate is calculated by integrating the outward, normal component of the normalized Poynting vector through a surface surrounding the antenna and the dipole emitter. The total decay rate takes the non-radiative losses into account, which are calculated by integrating the Ohmic losses across the volume of the antenna. Both energies require a normalization to the energy emitted by the same source into the same background material.

The antenna considered here has a width and thickness of 40 nm and a length of 127 nm. The length was tuned to be resonant at the emission wavelength of *λ* = 785 nm. To avoid unphysically sharp edges, we model the antenna as rounded with a radius of curvature of 10 nm. The considered gold properties are based on experimental data from^38^ for the permittivity of gold in the visible/near-infrared spectral region. The thickness of the ITO is set to 10 nm and its refractive index is taken from^39^. The glass substrate (SiO_2_) is modeled as a half-space and has a constant refractive index of *n* = 1.44. To avoid numerical artifacts, a minimum distance of 2 nm between the dipole emitter and the ITO or the antenna, respectively, is introduced.

## Additional Information

**How to cite this article**: Chen, H. *et al*. Manipulation of photoluminescence of two-dimensional MoSe_2_ by gold nanoantennas. *Sci. Rep.* **6**, 22296; doi: 10.1038/srep22296 (2016).

## Supplementary Material

Supplementary Information
