# Supplementary material for: Manipulation of photoluminescence of two-dimensional MoSe2 by gold nanoantennas
Source: Sci Rep. 2016 Feb 29;6:22296. doi: 10.1038/srep22296 (PMC4770425; doi:10.1038/srep22296)
Supplement: Supplementary Information [file srep22296-s1.pdf]

Manipulation of photoluminescence of two-dimensional $MoSe_2$ by gold nanoantennas

Supplementary Information

Haitao Chen[1], Jiong Yang[2], Evgenia Rusak[3], Jakob Straubel[3], Rui Guo[1], Ye Win Myint[2], Jiajie Pei[2], Manuel Decker[1], Isabelle Staude[1], Carsten Rockstuhl[3,4], Yuerui Lu[2], Yuri S. Kivshar[1], and Dragomir Neshev[1,*]

[1]Nonlinear Physics Centre, Research School of Physics and Engineering, Australian National University, Canberra, ACT 2601, Australia
[2]Research School of Engineering, College of Engineering and Computer Science, Australian National University, Canberra, ACT 0200, Australia
[3]Institute of Theoretical Solid State Physics, Karlsruhe Institute of Technology, 76131 Karlsruhe, Germany
[4]Institute of Nanotechnology, Karlsruhe Institute of Technology, 76021 Karlsruhe, Germany
[*]Dragomir.Neshev@anu.edu.au

Keywords: photoluminescence, 2D material, $MoSe_2$, nanoantennas, plasmonics

The photoluminescence (PL) of the samples were measured by commercial WiTec - alpha300S system in confocal microscope configuration, the setup of this system is shown in Figure S1.

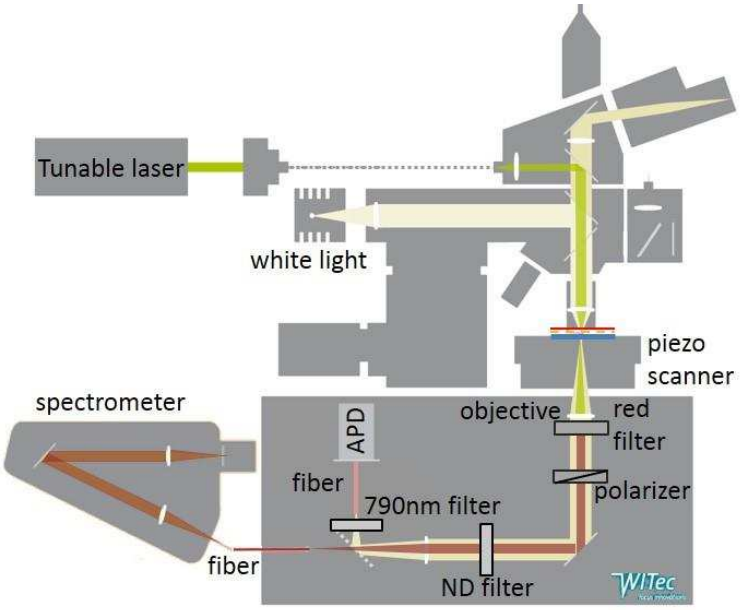

Figure S1. Photoluminescence measurement setup.The tunable laser covers a long wavelength range which enables us to change the excitation wavelength from 532 nm to 633 nm. The 790 nm filter generally refers to filters that passes wavelength around 790 nm while blocks the excitation wavelength, we used either a longpass filter cut at 630 nm or a 633 nm notch filter which blocks excitation laser.

To verify that excitation enhancement does not affect our results much, we measured the enhancement (quenching) effect with varying wavelength under same power, Figure S2 shows enhancement (quenching) effect dependence on excitation wavelength. The results here show that the enhancement (quenching) effect do not depend on excitation wavelength verifying that excitation does not contribute to the enhancement we observed here.

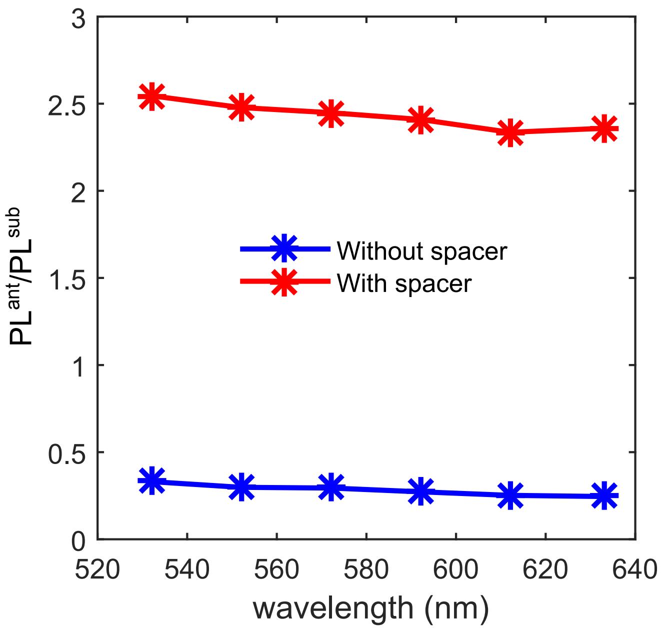

Figure S2. Antenna effects varying with excitation wavelength for both samples with a collection polarization along the gold particle long axis.

To confirm that the $MoSe_2$ flakes we used in experiments are monolayers, we also conducted Raman measurements. The Raman signal from two different parts of our sample are shown in Figure S3. The observed Raman peak at 240.8 cm^{-1} is consistent with results reported in Refs.[1,2] for monolayer $MoSe_2$, thus indicating that the flakes we used are monolayers. On the other hand, the results in the literatue show that the exact Raman peak of a monolayer can vary depending on the crystal quality and the method of exfoliation. Furthermore, the bilayer $MoSe_2$ Raman peak is relatively close to monolayer's (only different by $1 \sim 2$ cm^{-1}).[1,2] Therefore, the Raman signal could not exactly identify the number of layers of the $MoSe_2$ flakes. However, combined with the PL spectrum, shown in main text, we could conclude that the flakes we used in experiments are indeed monolayer $MoSe_2$.

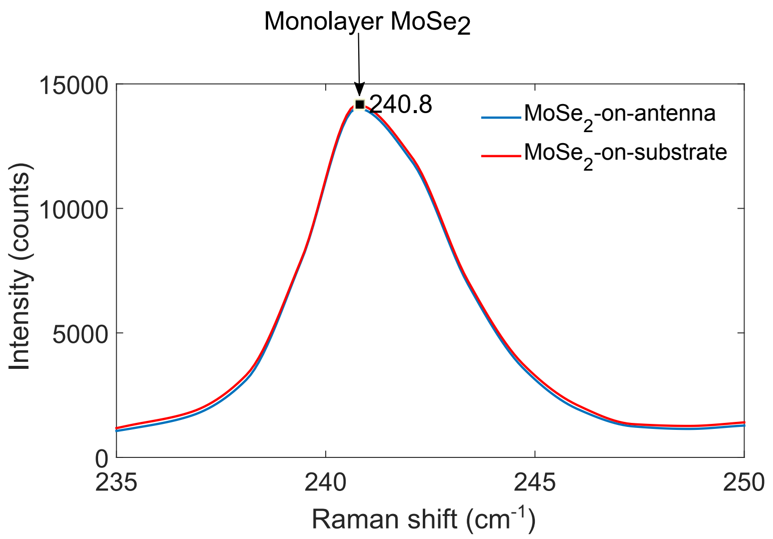

Figure S3. Raman signal of our $MoSe_2$ flake sample on different regions. Here we use 532 nm laser for excitation (laser power of around 280 μw), the signal is collected by 100x objective with numerical aperture of 0.9 and analysed by a grating spectrometer with 2400 l/mm.The original data resolution is not enough to give the exact peak position, the above curves are fitted by Piecewise cubic Hermite interpolation which preserves the shape of the curve well.

References

1. Tonndorf, P. *et al.* Photoluminescence emission and Raman response of monolayer MoS_2, $MoSe_2$, and WSe_2. *Opt. Express* **21**, 4908–4916 (2013). 1208.5864.
2. Tongay, S. *et al.* Thermally Driven Crossover from Indirect toward Direct Bandgap in 2D Semiconductors: $MoSe_2$ versus MoS_2. *Nano Lett.* **12**, 5576–5580 (2012).
